# Current Developments in the Use of FDM 3D-Printed Materials for Efficient Heat Transfer Applications

**DOI:** 10.3390/ma19132836

**Published:** 2026-07-03

**Authors:** Paweł Madejski, Ali Raza

**Affiliations:** Department of Power Systems and Environmental Protection Facilities, Faculty of Mechanical Engineering and Robotics, AGH University of Krakow, Al. Mickiewicza 30, 30-059 Kraków, Poland

**Keywords:** material extrusion, thermal properties, 3D-printed heat exchangers, thermal conductivity, transient plane source method

## Abstract

This work investigates the potential of additive manufacturing (AM) technologies for prototyping and developing functional components in thermal systems, with particular emphasis on thermal and mechanical performance. The study focuses on two complementary prototyping strategies: (i) the use of metal-filled polymer filaments in Fused Deposition Modeling (FDM), also known as Material Extrusion (MEX) according to ISO/ASTM 52900:2022, and (ii) a hybrid approach combining polymer 3D printing with conductive coating and electrochemical copper deposition. While metal-filled filaments provide a rapid and low-cost solution for early-stage prototyping, their mechanical properties remain similar to those of the polymer matrix, limiting their applicability in load-bearing structures. In contrast, the hybrid method enables the fabrication of hollow metallic geometries with improved thermal and electrical conductivity. This approach is more time-consuming and process-intensive and is therefore considered a subsequent stage in the prototyping workflow following initial MEX-based design iterations. Compared with conventional polymer-based MEX, several AM approaches enable the development and fabrication of fully metallic or metal-functional structures, including Powder Bed Fusion (PBF), Directed Energy Deposition (DED), and hybrid polymer–metal methods based on electroplating. Furthermore, understanding mechanical properties such as tensile strength is essential for assessing the applicability of AM materials in energy system components. The results contribute to bridging the gap between rapid prototyping and the implementation of advanced AM technologies in thermal-related applications.

## 1. Introduction

Additive manufacturing (AM) is the automated fabrication of 3D components layer by layer. AM techniques encompass numerous fabrication methods, including Stereolithography (SLA), Material Extrusion (MEX), Selective Laser Melting (SLM), and others [1,2]. MEX is one of the widely adopted AM techniques, mainly due to its cost-effectiveness and ease of fabrication. MEX has emerged as a prominent AM technique for fabricating complex structures from polymer materials [3,4]. It supports early-stage rapid prototyping and customized fabrication of components, leading to extensive applications across various sectors, including energy systems (particularly heat exchanger prototyping), aerospace, automotive, civil engineering, electrical and electronics, consumer products, and many more [5,6]. Several electronic applications require polymer materials that exhibit high thermal conductivity while also serving as excellent electrical insulators. Thus, it is crucial to understand the thermal performance of fabricated MEX components, which has attracted significant research and development interest. However, polymer-based materials inherently exhibit poor thermal stability, low thermal conductivity, and insufficient mechanical strength compared to metallic materials, consequently limiting their applicability in functional thermal and mechanical applications.

Madejski et al. [7] demonstrate that the infill geometry significantly affects the tensile performance of 3D-printed PLA. The Octet lattice achieved the highest strength (30.43 MPa) and notable ductility due to its stretch-dominated design. The Lines pattern excels at elongation and energy absorption, making it suitable for applications that require toughness. Cubic and Triangle infills offered higher stiffness but lower ductility, while Quarter-Cubic provided balanced, variable properties. Triangle patterns, with higher thermal conductivity, traded tensile strength for stiffness—ideal for heat-dissipating applications, such as electronic enclosures. Quarter-Cubic and Lines delivered better ductility and impact resistance, fitting insulation-focused designs. Octet offered a unique mix of strength and moderate thermal performance for multifunctional uses. This is thanks to the effective application of micro-computed tomography (micro-CT) combined with advanced image segmentation techniques to characterize internal porosity and defects in MEX 3D-printed PLA samples with different infill patterns [8]. To enhance the mechanical strength of the fabricated MEX parts, continuous fibers, including glass, carbon or Kevlar, can be incorporated into the polymer matrix, enabling their use at a functional scale [2].

To overcome the limitations associated with early-stage validation of a functional system, such as a heat exchanger, a metal-filled polymer filament can be fabricated using the MEX process. Although the results may not be directly comparable to those from a system made from fully metallic materials, this technique can assist in geometry optimization and preliminary appropriate material selection. A wide range of metal-filled polymer filaments is readily available on the market, including PLA filaments filled with copper, bronze, or steel from ColorFabb [9], as well as PLA filaments filled with 316L stainless steel, tungsten, high-carbon iron, titanium, and other metals offered by filament2print [10]. Thermal conductivity of the specimen was observed to be improved in proportion to the incorporated metal filler in matrix [11]. The thermal properties of PLA filled with copper, bronze, magnetic iron, and stainless steel were found to be higher than those of pure PLA [12].

The heat exchangers were fabricated using MEX process from PLA, metal-filled PLA (33% copper powder), and a high-thermal-conductivity (hi-k) nylon-based polymer with various geometrical configurations to evaluate the thermo-hydraulic performances of the heat exchangers. The results revealed that the hi-k material exhibited thermal performance close to that of the metal heat exchanger. Moreover, the bio-inspired configuration demonstrated high thermal performance with a low pressure drop, comparable to that of metal heat exchangers [13]. A tubular manifold microchannel heat exchanger was fabricated using the MEX process from ABS to enhance thermohydraulic performance while maintaining cost effectiveness. A copper fin tube was integrated into the fabricated design to ensure uniform flow distribution along the shell side under single-phase water flow conditions. The proposed MEX-fabricated design demonstrated efficient performance and shows strong potential as a promising option for industrial applications [14]. Baltic et al. [15] investigated two po-processing heat-treatment techniques, including annealing in salt and annealing in alabaster, to enhance the mechanical characterization of 3D-printed PETG specimens. The experimental results revealed that the properties, such as hardness, abrasive wear resistance, tensile properties, and impact resistance, were significantly improved compared to untreated specimens. The findings demonstrate that the treated specimens have strong potential for use in applications subjected to extreme environmental conditions. Moreover, the reinforcement of hybrid fillers in polymer materials has attracted considerable attention for improving material properties in various engineering applications. It has been reported that the incorporation of boron nitride nanosheets and silica nanoparticles into a polypropylene matrix significantly enhances the thermal, mechanical, rheological and tribological properties of composite material [16]. Novel heat exchanger designs were fabricated using laser powder bed fusion (LPBF) with an aluminum alloy (AlSi10Mg). The fabricated heat exchanger exhibited significantly enhanced heat transfer performance compared to conventional heat exchangers. However, a higher pressure drop was observed compared to traditional designs, attributed to increased surface roughness [17]. Innovative porous lattice air-cooled exchangers were manufactured using selective laser melting (SLM) process with an aluminum alloy (AlSi10Mg) to evaluate the thermo-hydraulic performance in a wind tunnel. The designs were based on Rhombi-Octet unit cells. The lattice structure exhibited a higher volumetric flow density than conventional heat exchangers and achieved approximately twice the heat transfer coefficient. As a result, their overall performance was significantly higher than that of conventional designs [18]. Experimental and numerical investigations of compact heat exchangers with different internal fin designs were conducted to examine their thermohydraulic performance. The structures were fabricated from stainless steel (316L) using LPBF. Overall, the study revealed that a small geometric modification significantly enhanced the thermo-hydraulic performance and indicated that the hydraulic diameter had a greater influence than surface roughness under laminar flow conditions [19]. Kus et al. [20] reported a detailed investigation on the thermo-hydraulic performance of compact heat exchangers incorporating a gyroid-type TPMS core structure. The heat exchangers were fabricated from stainless steel (316L) employing the LPBF technique. The results indicated that gyroid-based designs offer superior thermal performance compared to traditional designs; however, they require careful trade-offs between enhanced heat transfer and higher pressure drop. Zaki et al. [21] conducted an investigation into the design, fabrication, and performance assessment of an AM compact water-cooled refrigerant condenser (heat exchanger). The condenser was fabricated from aluminum alloy (AlSi10Mg) powder using laser powder bed fusion (LPBF). The overall findings revealed that the proposed design significantly enhanced the thermo-hydraulic performance and power density, providing a potential approach for developing advanced heat exchangers. Aljuhani et al. [22] presented a combined numerical and experimental investigation of the design, manufacturing, and thermo-hydraulic performance assessment of a novel vascular-based, bio-inspired heat exchanger, aiming to enhance heat transfer performance. The design was fabricated using LPBF from aluminum alloy (AlSi10Mg). The proposed design delivered superior thermal performance and compactness compared to traditional fin-tube and lattice-based heat exchangers. The findings revealed that the proposed design has promising potential for compact thermal applications; however, careful trade-offs between heat transfer enhancement and pressure drop are required.

The primary aim of this study is to provide a comprehensive overview of additive manufacturing (AM) techniques for heat transfer applications, particularly in the development of heat exchangers. Different AM techniques for metallic, polymer, and polymer–metal-filled materials are briefly discussed, with a focus on their applications in heat transfer systems. The limitations of each AM technique are also highlighted, with particular focus on Material Extrusion (MEX)-based materials. Furthermore, the study identifies suitable techniques and materials for early-stage prototyping of thermal systems before fabricating the final functional system, enabling a more cost-effective design process. In addition, selected thermal and experimental results related to metal-filled polymer materials are presented. Overall, this study provides researchers with a comprehensive overview of selecting appropriate AM techniques for both early-stage prototyping and final functional engineering systems, along with suitable material choices, thereby facilitating cost-effective development.

## 2. Background and Current State of 3D Printing Methods

In comparison to the presented MEX methods, several additive manufacturing technologies enable the production of fully metallic parts, including Directed Energy Deposition (DED), Powder Bed Fusion (PBF), Binder Jetting (BJT), and metal material extrusion (bound powder processes), either directly or after post-processing such as debinding and sintering.

Directed Energy Deposition is a process in which metallic powder is delivered through a nozzle into a melt pool generated by a focused energy source, typically a laser or plasma arc. The material is melted and solidifies immediately upon deposition, forming dense metallic structures. DED is particularly suitable for repairing high-value components and adding material to existing parts. Compared to other methods, it offers high deposition rates and flexibility in material usage. However, the process generally provides lower dimensional accuracy and surface quality than Powder Bed Fusion. It often requires subsequent machining to achieve final tolerances. DED is widely used in aerospace and energy sectors for large-scale components [23].

Powder Bed Fusion is one of the most established additive manufacturing technologies for metals. It involves selectively melting layers of fine metallic powder using a laser or electron beam. The process enables the production of highly complex geometries with excellent dimensional accuracy and fine feature resolution. Parts produced via PBF typically exhibit high density and good mechanical properties, comparable to wrought materials. The technology is widely applied in aerospace, medical implants, and precision engineering. However, it is relatively slow and requires support structures, as well as post-processing steps such as heat treatment. Powder handling and machine costs are also significant considerations [24].

Binder Jetting is a powder-based process in which a liquid binder is selectively deposited to join metal powder particles layer by layer. The resulting “green part” is fragile and requires post-processing. After printing, the part undergoes debinding and sintering to remove the binder and densify the structure. This process enables relatively fast production and does not require high-energy heat sources during printing. It is well suited for batch production and complex geometries. However, shrinkage during sintering must be carefully controlled. Final parts may have slightly lower density compared to PBF components. Despite this, BJT is increasingly used in industrial manufacturing [25].

Metal Material Extrusion, also known as bound powder extrusion, is based on a filament composed of metal powder mixed with a polymer binder. The material is deposited layer by layer similarly to MEX processes. The printed part, referred to as a “green part,” must undergo debinding to remove the polymer component. This is followed by sintering, which densifies the metal structure. The method is relatively accessible and cost-effective compared to other metal AM technologies. It is suitable for small to medium-sized components with moderate complexity. However, dimensional shrinkage and lower final density can be challenges. The process is often used for prototyping and small-scale production [26].

### 2.1. Material Extrusion (MEX) Method

Thanks to advances in the fabrication of MEX metal filaments, these materials are becoming more popular and widely available [27,28].

**Figure 1:**
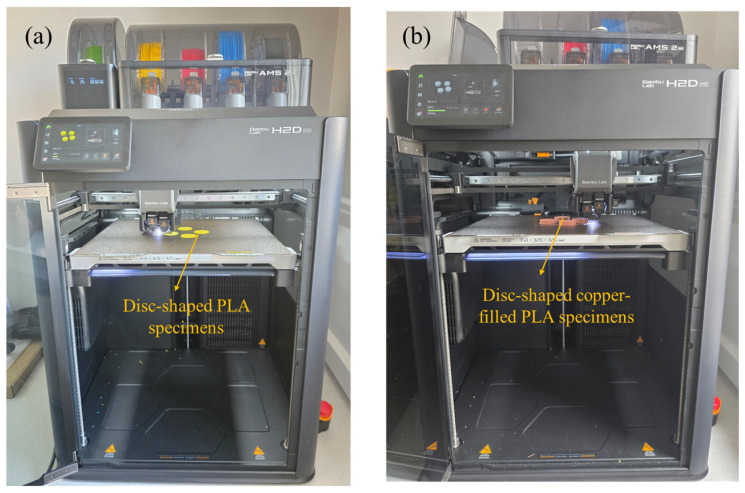
Fabrication process of PLA and PLA metal-filled specimens using Bambu Lab H2D Pro: (**a**) Pure PLA; (**b**) copper-filled PLA.

BASF Ultrafuse 316L filament combines greater design freedom with a low total cost of ownership, as reported by filament2print, Nigrán, Spain [10]. Three-dimensionally printed parts achieve their final properties, such as hardness and strength, through a BASF-developed deformation and sintering process that has become the industry standard. Filament Ultrafuse 316L is composed of 90% stainless steel by weight and 10% polymer, making it easily printable on many open-die desktop printers. The filaments from ColorFabb [9] are ready for printing. The fabrication of disc-shaped specimens (diameter: 32 mm; thickness: 13 mm), using PLA from Ultimaker and metal-filled PLA such as copper fill, bronze fill and steel fill containing approximately 80% metal particles by weight from ColorFabb on the Bambu Lab H2D Pro 3D printer (Bambu Lab, Shanghai, China), is illustrated in Figure 1.

The 3D printing parameters for the fabricated specimens are illustrated in Table 1. The thermal properties of these specimens were measured using the C-Therm Trident analyzer (C-Therm Technologies Ltd., Fredericton, NB, Canada). The filament densities and the average weights of the PLA and metal-filled PLA specimens are reported in Table 2. The metal-filled specimens were approximately three times heavier than those fabricated from regular PLA filament, due to the higher density of the metal-filled filaments. It was observed that the density of PLA copper fill was higher than that of the PLA bronze fill filament; however, weight of the 3D-printed PLA bronze fill specimens was found to be slightly higher than that of the PLA copper fill specimens. The lower weight of the PLA copper fill specimens may result from a higher level of internal porosity introduced during 3D printing. The specimens were fabricated using a line-infill pattern with 100% infill density. Tests have shown reliable results with all metal print heads. However, there is a possibility of clogging in a Tungsten Carbide nozzle during the fabrication of metal-filled PLA specimens due to improper temperature control and inappropriate nozzle size. A hotend with a Teflon or Tungsten Carbide insulator may exhibit greater adhesion to the filament, leading to clogging.

### 2.2. Hybrid Low-Cost Approach for Fabricating: Polymer Printing and Electroplating

The proposed hybrid manufacturing method combines polymer-based additive manufacturing with electrochemical metal deposition to produce hollow metallic structures. In the first stage, parts are fabricated using consumer-grade 3D printing technologies such as MEX [29,30] or Stereolithography (SLA) [31] with PLA or PVA. These techniques enable the creation of complex geometries, including internal channels that would be difficult to manufacture using conventional methods.

Since polymer materials are electrically insulating, the printed parts must be coated with a conductive layer. This is typically achieved using graphite-based conductive paints or EMI shielding coatings, which create a continuous electrically conductive surface. The coated part is then subjected to electroplating in a copper electrolyte bath (CuSO_4_/H_2_SO_4_), where copper is deposited under controlled current density until a layer thickness of approximately 0.2 mm is achieved [32].

In the final stage, the polymer core is removed to obtain a fully metallic structure. This can be performed either through thermal burnout (for PLA) or by dissolution in hot water (for PVA). The latter approach has proven more reliable for small-scale features such as narrow tubes. The result is a thin-walled metallic component that replicates the geometry of the original polymer model. One of the main advantages of this method is its ability to produce highly complex and compact geometries at relatively low cost. Unlike metal additive manufacturing technologies such as Powder Bed Fusion (PBF) or Directed Energy Deposition (DED), this approach does not require expensive industrial equipment. Instead, it relies on widely available desktop 3D printers and relatively simple electroplating setups.

## 3. Perspective

### 3.1. Limitations and Challenges of Metal-Filled Filament in MEX Method

The use of metal-filled filaments, such as copper, in MEX presents significant limitations compared to true metal additive manufacturing processes. These materials consist of polymer matrices (typically PLA) loaded with metal particles and were primarily intended for aesthetic rather than structural applications. Importantly, they are not equivalent to sinterable metal feedstocks used in Metal Injection Molding (MIM) or bound metal extrusion systems.

**Figure 2:**
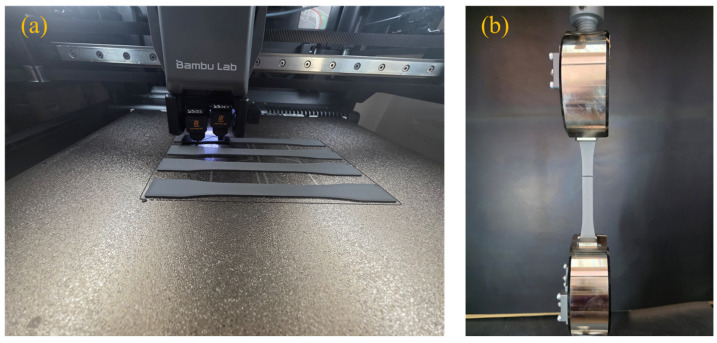
Fabrication of PLA steel-filled specimens for tensile testing (**a**); Tensile testing of PLA steel-filled specimens (**b**).

A key limitation is that such filaments do not undergo proper densification into solid metal through standard desktop post-processing. Unlike specialized metal filaments, steel-filled materials cannot be transformed into fully metallic parts via conventional debinding and sintering. Achieving true metal properties would require dedicated feedstocks and industrial-grade thermal processing, which are not applicable in this case. As a result, printed parts retain a composite structure rather than becoming homogeneous metal. Mechanical performance is another major constraint. The properties of printed components are largely determined by the polymer binder, resulting in behavior similar to that of PLA. This results in relatively low strength, brittleness, and limited thermal resistance compared to metals. The fabrication of pure PLA and steel-filled PLA specimens with a 100% infill line pattern (±45° infill angle) was carried out using the Bambu Lab H2D Pro 3D printer, according to the ASTM D638 Type I standard [33] for tensile testing. The printed specimens were tested using an AGX-V2 Universal Testing Machine (UTM) manufactured by Shimadzu, Kyoto, Japan. The average ultimate tensile strength of pure PLA specimens was found to be approximately four times higher than that of steel-filled PLA, i.e., 55 MPa for PLA and 15 MPa for steel-filled PLA. The fabrication of PLA steel-filled specimens (Figure 2a) and their testing using a UTM (Figure 2b) are shown in Figure 2. Consequently, such parts are suitable mainly for decorative purposes, including visual prototypes, jewelry, or artistic objects, but not for load-bearing or functional engineering applications.

Metal-filled MEX filaments for now do not provide a viable pathway to fully metallic components. They should be treated as polymer-based composites with enhanced visual appearance rather than as true metal manufacturing solutions. Despite the limitations associated with metal-filled filaments in MEX, these materials can still play a valuable role in engineering practice, particularly in rapid prototyping. Although they do not provide fully metallic mechanical properties, their ease of processing, low cost, and compatibility with consumer-grade equipment make them highly attractive for early-stage design exploration.

**Figure 3:**
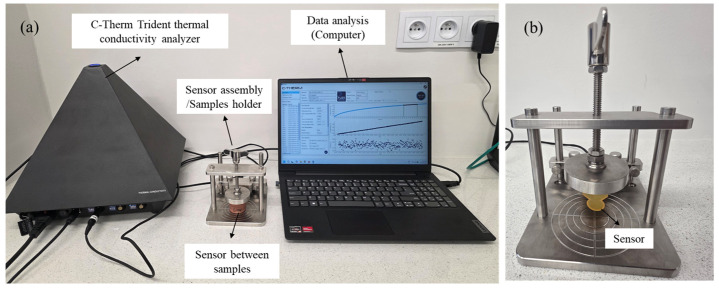
Experimental setup for TPS method: Overall experimental setup (**a**); View of the sensor assembly and sample holder (**b**).

One important application area is the prototyping of components for heat or mass transfer, especially those with complex internal geometries, such as channels, lattices, or compact heat exchangers. The ability to quickly fabricate such structures using MEX allows for iterative design and testing cycles that would be significantly more expensive and time-consuming with fully metallic manufacturing methods. While the thermal performance of metal-filled polymers is lower than that of bulk metals, it is still superior to that of pure polymers, making them suitable for comparative and preliminary studies. A key aspect in this context is the characterization and understanding of the effective thermal properties of printed metal–polymer composites, such as thermal conductivity. By experimentally determining these properties, it becomes possible to perform meaningful thermal analyses and validate numerical models (e.g., CFD simulations) at an early stage. This enables engineers to assess design concepts, optimize geometries, and identify potential performance issues before committing to costly metal production processes. An additional advantage of such materials is the possibility of experimentally determining their effective thermal properties using transient measurement techniques, such as the Transient Plane Source (TPS) or Modified Transient Plane Source (MTPS) methods. The experimental setup for the TPS method is presented in Figure 3. These techniques enable the evaluation of key parameters including thermal conductivity, thermal diffusivity, and thermal effusivity [34]. The thermal conductivity of metal-filled PLA (copper fill, Bronze fill and steel fill) was found to be more than twice that of pure PLA. TPS-based methods typically involve a sensor acting as both a heat source and a temperature monitor, placed between the sample surfaces, whereas MTPS allows single-sided measurements with minimal sample preparation. The relatively fast measurement time and good repeatability make these methods well suited for characterizing 3D-printed composites. Obtaining accurate thermal property data is essential for correlating experimental results with numerical simulations and for scaling prototype performance to fully metallic designs.

Furthermore, such materials enable rapid, accessible prototyping workflows, allowing multiple design variants to be produced and tested in a short time. This significantly reduces development risk and supports more informed decision-making. Once a design has been validated experimentally using metal-filled MEX prototypes, it can be more confidently translated into fully metallic components manufactured using advanced techniques such as Powder Bed Fusion or Directed Energy Deposition. In summary, while metal-filled MEX materials are not suitable for producing final functional metal parts, they represent a practical and efficient tool for prototyping complex geometries and conducting preliminary thermal investigations. Their use can bridge the gap between conceptual design and high-performance metal manufacturing, ultimately enabling more efficient, cost-effective product development.

### 3.2. Limitations and Challenges of Hybrid Low-Cost Approach

In analogy to metal-filled MEX materials, the hybrid polymer–metal manufacturing approach also offers significant potential for experimental determination of effective thermal properties. Due to the presence of a continuous metallic layer (e.g., electroplated copper), the thermal behavior of such structures is considerably closer to that of real metallic components than in the case of polymer-based composites. This makes the method particularly attractive for preliminary thermal investigations of functional geometries, such as heat exchangers or flow devices. However, it should be emphasized that this approach is more time-consuming and process-intensive than the conventional MEX technique, as it involves additional steps such as surface preparation, electroplating, and polymer removal. For this reason, it is best considered as a subsequent stage in the prototyping workflow, following initial design iterations performed using standard MEX techniques. In such a workflow, simple and rapid MEX prototypes can be used for early geometry validation, while the hybrid method enables more advanced testing under conditions that better approximate the thermal behavior of fully metallic components. However, it should be noted that the accuracy of thermal characterization depends strongly on the quality of the electroplated layer. Variations in coating thickness, defects, or incomplete coverage may lead to anisotropic or non-uniform thermal behavior. Despite these limitations, the hybrid method provides a valuable intermediate step between polymer prototyping and high-cost metal manufacturing, enabling more reliable experimental studies and reducing development risk in early design stages. The method is particularly suitable for research and development applications, including rapid prototyping, validation of computational fluid dynamics (CFD) models, and early-stage design testing. Additionally, it enables the fabrication of hollow metallic structures, which are difficult to achieve using conventional MEX alone due to material limitations. Compared to pure MEX technique, the hybrid approach significantly enhances thermal and electrical conductivity, making it more suitable for functional applications such as heat exchangers.

Despite its advantages, the method also has several limitations. The quality and uniformity of the electroplated copper layer are not yet fully optimized, which may result in uneven thickness or defects. Mechanical properties of the final component may be inferior to those produced by fully metallic processes such as PBF, due to potential porosity or weak interlayer bonding. Another challenge is removing the polymer core. Thermal burnout can lead to structural damage, especially in small or thin-walled features, while dissolution processes may be time-consuming. Additionally, the multi-step nature of the method increases process complexity and introduces potential sources of error at each stage.

In comparison with established metal additive manufacturing technologies such as PBF, DED, or Binder Jetting (BJT), the hybrid method is significantly more accessible and cost-effective. Nevertheless, it does not yet achieve the same level of material density, mechanical strength, or process reliability. Therefore, it is best suited for prototyping and experimental applications rather than high-performance industrial components. The hybrid polymer–metal manufacturing approach represents a promising alternative for producing complex metallic geometries at low cost. By combining the design flexibility of polymer 3D printing with the functional properties of metals, it bridges the gap between rapid prototyping and metal manufacturing. However, further optimization is required to improve coating quality, process reliability, and mechanical performance in comparison to established metal additive manufacturing technologies.

## 4. Future Directions

Future research is required to improve the AM of metal-filled polymers, such as PLA with copper, bronze or silver, using MEX techniques. Metal-filled PLA exhibits thermal conductivity values more than twice those of pure PLA; however, its ultimate strength is approximately four times lower. Therefore, there is a significant need to enhance the mechanical strength of the filament so that it can be reliably used for early-stage prototyping prior to the fabrication of final functional thermal systems.

The MEX technique is considerably more economical than hybrid manufacturing approaches and metal AM techniques. Although MEX-fabricated systems do not provide performance comparable to fully metallic systems, they offer a cost-effective, straightforward solution for fabricating early-stage prototypes for thermal applications.

During the manufacturing of metal-filled PLA specimens, clogging in the printer nozzle was observed due to improper temperature control, material flow conditions, and an inappropriate nozzle size. This issue was experienced by authors during the printing of specimens using a Bambu Lab H2D Pro 3D printer with PLA filled with copper, bronze or silver. Therefore, proper nozzle selection is critical and must be aligned with the metal particle content of the filament. The authors used a 0.6 mm nozzle due to the high metal particle content in the PLA filament (approximately 80% by weight). The standard 0.4 mm nozzle was found to be unsuitable due to frequent clogging, which compromised the printing process.

Hybrid polymer-metal manufacturing techniques typically provide higher mechanical and thermal performance than MEX-based early-stage prototypes, with results closer to those of metallic systems. However, these processes are time-consuming and expensive. Further improvement in coating quality and uniformity, as well as in adhesive layer control during electroplating, can be pursued to achieve mechanical and thermal performance comparable to a functional system fabricated using DED, PBF, BJT, and metal material extrusion for thermal applications.

## 5. Conclusions

This study addresses the suitability and limitations of additive manufacturing techniques, with a particular focus on MEX-based materials, including pure polymers and metal-filled polymers, for thermal engineering applications.

Metal-filled polymers, such as PLA copper- and steel-filled components fabricated using the MEX technique, offer a practical and economical solution for the rapid prototyping of engineering systems intended for preliminary thermal investigations.

Compared to pure polymers, metal-filled polymer composites exhibit improved thermal properties, with approximately twice the thermal conductivity. However, the presence of metal particles generally increases material brittleness, resulting in approximately 3 times lower mechanical strength than that of pure polymers. Despite improvements in thermal properties, the overall thermal performance and mechanical properties remain significantly inferior to those of conventional metallic materials. As a result, these limitations restrict their widespread use in industrial applications. However, these materials can still play a valuable role in engineering practice, particularly in rapid prototyping, and are highly attractive for early-stage design exploration.

Metal additive manufacturing technologies, such as DED, PBF, BJT, and others, enable the manufacture of fully metallic components with excellent thermal properties and high mechanical strength, comparable to those of conventional metallic materials. These techniques are capable of manufacturing complex geometries with high dimensional accuracy. However, they are typically associated with high equipment and feedstock costs, longer processing times, and complex post-processing requirements, such as machining, heat treatment, and debinding. Despite these issues, these techniques are widely applied in the aerospace, energy, and other advanced engineering sectors.

In contrast, hybrid manufacturing approaches offer a promising alternative that integrates the MEX technique with metallization processes. In this approach, the geometry is initially fabricated from a polymer using the MEX technique, then processed with a conductive coating and electroplating to produce a functional metallic geometry. Although the resulting geometries do not attain mechanical strength comparable to that of metallic materials, their thermal performance is considerably closer to that of metallic materials. Consequently, it is well suited for prototyping and initial thermal investigations of functional geometries, such as heat exchangers or flow devices rather than for advanced industrial applications.

The components produced using MEX with metal-filled PLA do not provide realistic thermal and mechanical performance for industrial use; however, they are suitable for early-stage design prototyping intended for thermal investigations. In comparison, objects produced through the hybrid approach exhibit thermal and mechanical performance close to that of real metallic components. Once the design is validated using the hybrid approach, it can be further manufactured through advanced metal manufacturing techniques.

Future developments in materials, optimization of manufacturing techniques, and integration with numerical modeling can significantly improve the applications of additive manufacturing in thermal engineering. Moreover, these developments are expected to allow a transition from prototyping toward real industrial thermal applications.

## Figures and Tables

**Table 1:** Three-dimensional printing parameters for PLA and metal-filled PLA specimens.

Materials	PLA	Metal-Filled PLA
Nozzle diameter	0.4 mm	0.6 mm
Extrusion temperature	220 °C	215 °C–230 °C
Heated bed	55 °C	55 °C–60 °C
Layer height	0.2 mm	0.4 mm
Printing speed	300 mm/s	50 mm/s
Infill pattern	Line	Line
Infill density	100%	100%

**Table 2:** Density and weights of disc-shaped specimens fabricated with metal-filled PLA and pure PLA.

Materials	Density (g/cm^3^)	Weight (g)
PLA	1.24	12.51
PLA Copper Fill	4	36.95
PLA Bronze Fill	3.9	37.13
PLA Steel Fill	3.13	31.08

## Data Availability

Data sharing is not applicable to this article as no new data were created or analyzed in this study.
